# The Prevalence of Coronary Artery Disease in Bicuspid Aortic Valve Patients: An Overview of the Literature

**DOI:** 10.1055/s-0044-1785190

**Published:** 2024-05-02

**Authors:** Onur B. Dolmaci, Tijmen L. Hilhorst, Arjan Malekzadeh, Bart J.A. Mertens, Robert J.M. Klautz, Robert E. Poelmann, Nimrat Grewal

**Affiliations:** 1 Department of Cardiothoracic Surgery, Leiden University Medical Center, Leiden, The Netherlands; 2 Department of Cardiothoracic Surgery, Amsterdam University Medical Center, Amsterdam, The Netherlands; 3 Medical Library, Amsterdam University Medical Center, Location University of Amsterdam, Amsterdam, Netherlands; 4 Department of Statistics, Leiden University Medical Center, Leiden, Netherlands; 5 Institute of Biology, Leiden University, Sylvius Laboratory, Leiden, Netherlands; 6 Department of Cardiology, Leiden University Medical Center, Leiden, The Netherlands; 7 Department of Anatomy and Embryology, Leiden University Medical Center, Leiden, The Netherlands

**Keywords:** aortic valve stenosis, atherosclerosis, bicuspid aortic valve, coronary artery disease, tricuspid aortic valve, coronary revascularization

## Abstract

The prevalence of coronary artery disease (CAD) in bicuspid aortic valve (BAV) patients is a debatable topic. Several studies have indicated that BAV patients have a lower prevalence of CAD compared with patients with a tricuspid aortic valve (TAV), but the effects of age and gender have not always been considered. This systematic review provides an overview of articles which report on CAD in BAV and TAV patients. Searches were executed in April 2021 and January 2022 according to the PRISMA (Preferred Reporting Items for Systematic reviews and Meta-analyses) guidelines in three online databases: Medline, Embase, and Scopus. Screening and data extraction was done by two investigators separately. Primary and secondary outcomes were compared between BAV and TAV patients; a fixed effects model was used for correcting on confounders. Literature search yielded 1,529 articles with 44 being eligible for inclusion. BAV patients were younger (56.4 ± 8.3 years) than TAV patients (64 ± 10.3 years, *p* < 0.001). All CAD risk factors and CAD were more prevalent in TAV patients. No significant difference remained after correcting for age and gender as confounders. BAV patients have a lower prevalence of CAD and CAD risk factors compared with TAV patients. However, when the age differences between both groups are considered in the analyses, a similar prevalence of both CAD and CAD risk factors is found.

## Introduction

A bicuspid aortic valve (BAV) is the most common congenital cardiac anomaly, with a prevalence of 1 to 2% in the general population. 1 2 Early embryonic defects are held responsible for the development of a BAV and are also associated with the development of thoracic aortopathy in these patients. 3 4 Besides the high risk for developing thoracic aortopathy, 5 BAV patients are also at risk of developing aortic valve diseases such as an aortic valve stenosis. 1 2 Although both BAV and tricuspid aortic valve (TAV) patients may develop these diseases, the risk in BAV patients is considered much higher with an additional earlier onset of these alterations compared with patients with a TAV. 6

Aside from the differences in risk and onset of the aortic valve disease, BAV and TAV patients also show differences in pathophysiology and population characteristics, which is best seen in aortic valve stenosis patients. Traditionally, cardiovascular aging (i.e., wear and tear) was considered as the sole contributor to aortic valve calcification (i.e., stenosis). However, recent studies have now shown an important role of cardiovascular risk factors, such as hypertension, hypercholesterolemia, smoking, age, and male sex, in the development of an aortic valve stenosis. 7 8 9 10 11 This multifactorial pathophysiology, which is considered the atherosclerotic disease spectrum, is also the underlying cause of the association of an aortic valve stenosis with coronary artery disease (CAD). 7 12 13 Although these new observations are true for TAV patients, BAV patients do not fit the same profile as TAV patients and the exact pathogenesis of aortic valve stenosis in BAV patients remains unclear. While carrying a higher risk for aortic valve stenosis, the prevalence of cardiovascular risk factors and CAD is found significantly lower in BAV compared with that of TAV patients. 12 13 Furthermore, less calcification and atherosclerotic plaque formation is found in the thoracic aorta of BAV patients, which led to the hypothesis that BAV patients have a lower atherosclerotic disease burden compared with TAV patients. Only a few studies have directly investigated atherosclerosis in BAV patients through imaging (e.g., coronary angiography or computed tomography) or histology. Our knowledge of the role and prevalence of atherosclerosis in BAV patients therefore remains scarce. The literature regarding this subject is also inconsistent, with some sources even suggesting an increased risk for atherosclerosis in BAV individuals. 14 Since direct investigations of atherosclerosis are rare, clinical CAD and coronary revascularization (both indirect markers of atherosclerosis) are often used to compare and evaluate the atherosclerotic disease burden in BAV patients.

This review provides an overview of the studies that reported on CAD in BAV patients. Furthermore, comparisons will be made with TAV patients and the prevalence of cardiovascular risk profiles will be provided as secondary outcomes.

## Methods

### Study Objectives

The purpose of this analysis is to provide an overview of studies reporting on the prevalence of CAD and CAD risk factors in BAV and TAV patients. Primary outcomes were a prior myocardial infarction, prior percutaneous coronary intervention (PCI), prior coronary artery bypass grafting (CABG) and concomitant CABG. Secondary outcomes were the CAD risk factors, which included hypertension, hypercholesterolemia, and diabetes mellitus.

### Search Strategy and Study Selection

Two delimited searches were executed in April 2021 and January 2022, in line with the PRISMA (Preferred Reporting Items for Systematic reviews and Meta-Analyses) guidelines. 15 Literature search was performed using online databases (Medline [Ovid], Embase [Ovid], and Scopus). The searches contained terms for bicuspid and TAVs, coronary revascularization (e.g., PCI and CABG), myocardial ischemia, and CAD. The search strategy was not restricted by the year of publication. Studies that could not be translated reliably, case reports, reviews, and animal studies were excluded (see Supplementary File for full search strategy , available in online version only). Two authors (O.B.D. and T.L.H.) screened all articles independently based on title and abstract using Rayyan. 16 Included articles were then reviewed in full text. In case of conflict in inclusion, discordances were discussed and resolved.

### Data Extraction

All studies reporting presence of CAD (including coronary revascularization through CABG or PCI) in BAV and in TAV patients were included and evaluated in this analysis. If a paper was considered eligible, data were extracted. Extracted data included: sample size subdivided into BAV and TAV, demographics, history of CAD (prior myocardial infarction, prior PCI, prior CABG), concomitant CABG, presence of CAD (through coronary imaging), risk factors for CAD (hypertension, hypercholesterolemia, diabetes mellitus, tobacco usage, body mass index), and mortality.

### Statistical Analysis (and Risk of Bias Assessment)

Data are presented as absolute number of cases with percentages, means, and standard deviation (reported as mean ± standard deviation) in continuous variables with a normal distribution and as median with the interquartile range in continuous variables without a normal distribution. Normality tests, skewness, and kurtosis were performed for all variables. Normally distributed continuous data were compared using the *t* -test. In continuous variables without a normal distribution, the Mann–Whitney U test was used, and the Fisher's exact test was used for categorical data. A fixed effects model was developed to correct for the differences in age and gender between the BAV and TAV groups. A *p* -value of <0.05 was considered to be significant. All statistical analyses were conducted using IBM SPSS for Windows version 25.0.

## Results

### Literature Search and Outcome

**Fig. 1:**
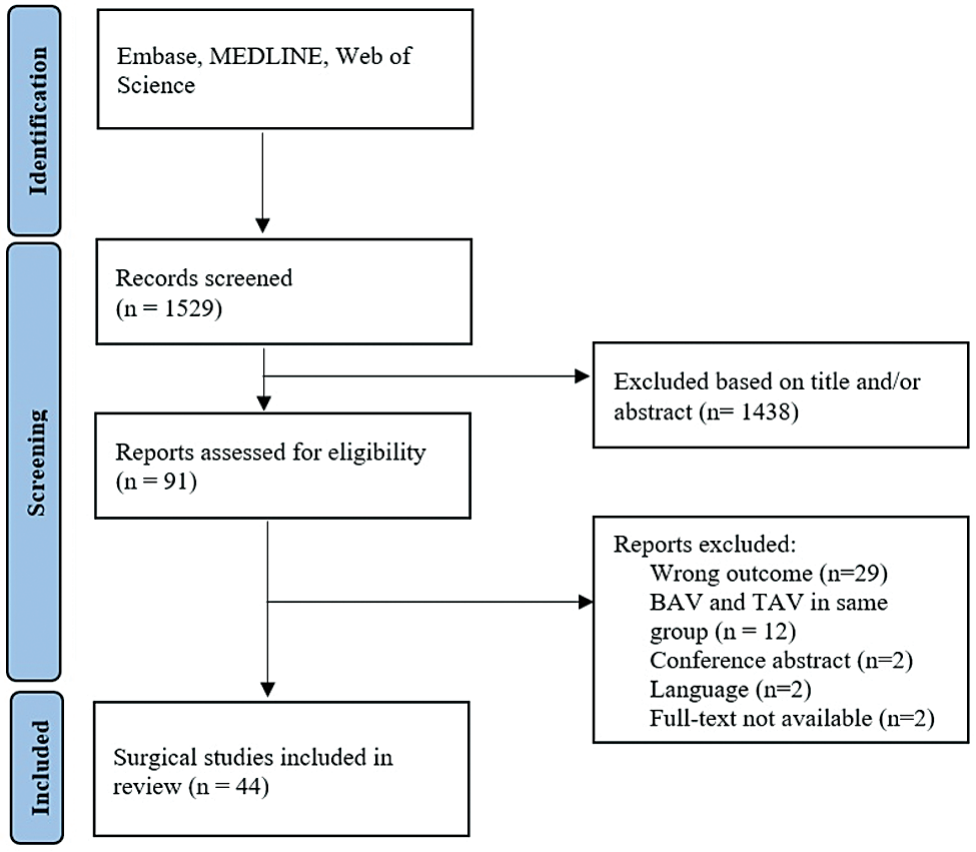
Selection flowchart.

**Fig. 2:**
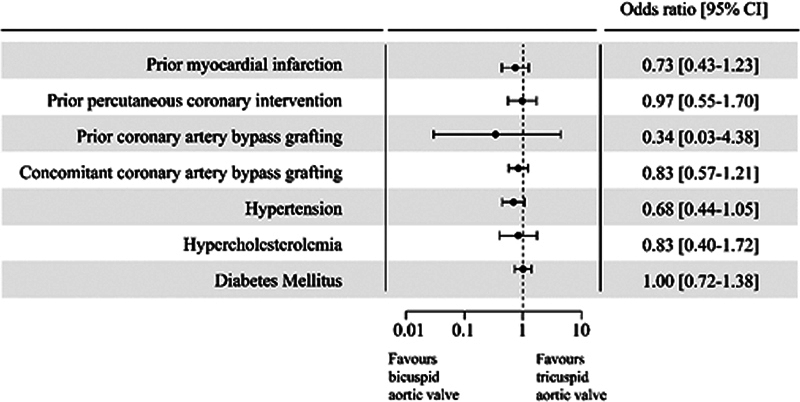
Forest plots of the corrected analyses. Figure shows the forest plots of the corrected analyses for each outcome. All outcomes show to be equally prevalent between both groups after correcting for the age and sex differences between the BAV and TAV groups. BAV, bicuspid aortic valve; CI, confidence interval; TAV, tricuspid aortic valve.

The initial literature search yielded 1,529 studies. Fig. 1 shows the overview of the selection process of this systematic review. After selection, a total of 44 articles were eligible for inclusion in this systematic review. The articles reported data on a sum of 60,695 patients, of which 19,934 (32.8%) were patients with a BAV. The articles mainly reported on male subjects ( *n* = 41,471, 68.3%) in both groups with a mean age of 60.2 years (± 10 years). BAV patients were younger (56.4 ± 8.3 years) compared with TAV patients (64 ± 10.3 years, *p* < 0.001). An overview of the outcomes are provided in Tables 1 , 2 and in Fig. 2 .

**Table 1:** Overview of outcomes

	Bicuspid aortic valve		Tricuspid aortic valve	
Medical history	Number of patients with reported outcome (%)	Total patients in studies	Number of patients with reported outcome (%)	Total patients in studies
Prior myocardial infarction	101 (6.9)	1,467	667 (13.2)	5,037
Prior percutaneous coronary intervention	409 (2.9)	14,247	1,642 (5.6)	29,166
Prior coronary artery bypass grafting	151 (1.0)	14,416	932 (3.4)	27,173
Concomitant coronary artery bypass grafting	1,095 (23.1)	4,746	4,486 (39.5)	11,349
Hypertension	10,045 (57.2)	17,560	24,847 (70.5)	35,247
Hypercholesterolemia	730 (27.4)	2,660	2,580 (36.5)	7,061
Diabetes mellitus	2,148 (11.7)	1,8317	6,316 (16.3)	38,703

Abbreviation: CAD, coronary artery disease.

Note: The absolute (uncorrected) prevalence of CAD and CAD risk factors per group and the total number of included patients, of which these outcomes were reported.

**Table 2:** Fixed effects model (primary outcomes)

Medical history	Coefficient	Standard error	*p* -Value
**Prior myocardial infarction**	−0.318	0.24	0.215
**Prior percutaneous coronary intervention**	−0.032	0.24	0.968
**Prior coronary artery bypass grafting**	−1.094	1.15	0.366
**Concomitant coronary artery bypass grafting**	−0.192	0.19	0.311

Note: Evaluation of the primary outcomes using a fixed effects model.

## Coronary Artery Disease

### Prior Myocardial Infarction

Nine studies 12 13 17 18 19 20 21 22 23 reported on the prevalence of prior myocardial infarction, which included a total of 6,504 patients. Myocardial infarction was reported in 768 (11.8%) of the total group. Of all included BAV patients, 6.9% had a prior myocardial infarction (101 of 1,467 included patients) versus 13.2% of TAV patients (667 of 5,037 included patients), which was a significant difference ( *p* < 0.001). No significant difference remained after correcting for the age and gender differences between both groups (odds ratio [OR] = 0.73 [95% confidence interval, CI = 0.43–1.23]; *p* = 0.215).

### Prior Percutaneous Coronary Intervention

Six studies 12 13 18 19 21 24 reported on the prevalence of a prior PCI, which included a total of 43,413 patients. A PCI was performed in the past in a total of 2,051 (4.7%) patients. A prior PCI was reported in 409 (2.9%) of 14,247 BAV patients and in 1,642 (5.6%) of 29,166 TAV patients ( *p* < 0.001). After correcting for age and gender, a nonsignificant difference was seen between both groups (OR = 0.97 [95% CI = 0.55–1.70]; *p* = 0.898).

### Prior Coronary Artery Bypass Grafting

Seven studies 12 13 20 21 24 25 26 reported on the prevalence of a prior CABG, which included a total of 41,589 patients. Within this group, 1,083 (2.6%) patients had a CABG in their medical history. The prevalence in the BAV group was 151 (1%) of 14,416 and 932 (3.4%) of 27,173 in the TAV group ( *p* < 0.001). However, after correction for age and gender, the difference became nonsignificant (OR = 0.34 [95% CI = 0.03–4.38]; *p* = 0.366).

### Concomitant Coronary Artery Bypass Grafting

Twenty-five studies 7 12 13 18 20 23 26 27 28 29 30 31 32 33 34 35 36 37 38 39 40 41 42 43 44 reported on the prevalence of a concomitant CABG, which included a total of 16,095 patients. A concomitant CABG was performed in a total of 5,581 (34.7%) patients. These included 1,095 (23.1%) of 4,746 BAV patients, 4,486 (39.5%) of 11,349 TAV patients ( *p* < 0.001). After correction for age and gender, the difference between both groups became nonsignificant (OR = 0.83 [95% CI = 0.57–1.21]; *p* = 0.311).

## Cardiovascular Risk Factors

### Hypertension

Thirty-five studies 7 12 13 17 18 19 20 21 22 23 24 25 26 28 29 32 33 34 36 39 40 45 46 47 48 49 50 51 52 53 54 55 56 57 58 reported on the prevalence of hypertension, which included 52,807 patients. Hypertension was present in a total of 34,892 (66.1%) patients. These included 10,045 (57.2%) of 17,560 BAV patients and 24,847 (70.5%) of 35,247 TAV patients ( *p* < 0.001). After correcting for age and gender, the difference became nonsignificant (OR = 0.68 [95% CI: 0.44–1.05]; *p* = 0.082).

### Hypercholesterolemia

Twenty-three studies 12 13 17 18 20 21 23 25 26 28 29 33 39 40 45 47 49 50 51 55 56 57 59 reported on the prevalence of hypercholesterolemia, which included a total of 5,240 patients. Within this group, 3,310 (63.2%) had hypercholesterolemia. These included 730 (27.4%) of 2,660 BAV patients and 2,580 (36.5%) of 7,061 TAV patients ( *p* < 0.001). After correcting for age and gender, these differences became nonsignificant (OR = 0.83 [95% CI = 0.40–1.72]; *p* = 0.602).

### Diabetes Mellitus

Thirty-five studies 7 12 13 17 18 19 20 21 22 23 24 25 26 28 29 31 32 33 34 36 39 40 45 46 47 48 49 50 51 53 55 56 57 58 59 reported on the prevalence of diabetes mellitus, which included a total of 57,020 patients. In a total of 8,464 (14.8%) patients within this group diabetes mellitus was present. These included 2,148 (11.7%) of 18,317 BAV and 6,316 (16.3%) of 38,703 TAV patients ( *p* < 0.001), which was nonsignificant after correction for age and gender (OR = 1.00 [95% CI = 0.72–1.38]; *p* = 0.989).

## Discussion

This systematic review aimed to provide an overview of all articles that reported on the prevalence of CAD and risk factors for CAD in BAV patients, and to compare these data with those of TAV patients. These results showed a lower prevalence of CAD and CAD risk factors in BAV patients. However, when corrected for the difference in age between the BAV and TAV patients, no significant differences in the prevalence of both CAD and CAD risk factors remained.

Comparisons between BAV and TAV patients have always been complicated due to the differences in age between both groups at the time of surgery, since BAV patients are on average 7 to 10 years younger than TAV patients at the time of surgery. 6 Especially when focusing on a topic like the prevalence of atherosclerosis, in which age is an important contributing factor in the pathophysiology, it is crucial to consider age as an important confounder. This is also highlighted in the current study, in which all significant differences disappeared after correcting for the age differences. Similar results were seen in a previous systematic review in which age was also an important confounder. 60 This indicates no clinical differences in CAD and coronary revascularization between BAV and TAV patients. Although not significantly different, the prevalence of CAD risk factors was high in both groups, indicating that an individual approach for treating these comorbidities is important for both groups. Clinicians should especially focus on the treatment of hypertension in BAV patients, as both hypertension and a BAV are important risk factors for developing an aortic dissection.

In this review CAD was chosen to study as a marker for atherosclerosis, since papers that directly investigate the presence of atherosclerotic plaque formation (e.g., with coronary imaging or histopathologically) are scarce. 12 13 It is important to point out that CAD is an end-stage disease and coronary revascularization is only advised in patients with coronary stenosis of more than 70%. 61 Only studying CAD as a marker for atherosclerosis would therefore exclude the larger portion of patients with coronary sclerosis that causes less than 70% coronary obstruction.

Previous studies that used different modalities to directly investigate the presence of atherosclerotic plaque formation in BAV patients (e.g., with coronary angiography, computed tomography, and histopathology) indicated that BAV patients have a lower prevalence of CAD (and atherosclerotic plaque formation) when compared with age- and sex-matched TAV patients. 12 13 As mentioned earlier, differences in aortic wall composition between BAV and TAV patients could be an explanation for the lower tendency to develop atherosclerosis in BAV patients. Histopathological studies have revealed a thinner intimal layer of the aortic wall and a phenotypical switch defect of vascular smooth muscle cells characteristic for BAV patients. 3 62 63 Since the vascular smooth muscle cells are important contributors to atherosclerotic plaque formation and the plaques develop in the intima, the abovementioned vascular defects could complicate the formation of plaques within this layer and therefore result in a lower tendency for developing atherosclerosis.

Based on the results of our studies, no conclusions can be drawn about the prevalence of general atherosclerosis in BAV patients. However, this study did show a comparable prevalence of CAD between BAV and TAV patients, as an indirect measure of atherosclerosis. This implies that whether or not a difference in atherosclerosis is present between both groups, it does not cause significant differences clinically regarding CAD and coronary revascularization. This study endorses that age is an important factor in the development and presence of CAD, which could contribute to lesser findings in the preoperative workup of BAV patients. Less invasive coronary imaging techniques (such as computed tomography) could be considered as a good first step in preoperative BAV patients with a low cardiovascular risk profile (e.g., no CAD risk factors and a low age) instead of a traditional coronary angiography.

## Limitations

As pointed out before, this review only focused on late (clinical) outcomes of atherosclerosis (CAD with significant coronary occlusion). The conclusions drawn out of this study therefore are only based on the late stages of atherosclerosis and do not include patients with coronary stenosis, which is not significant (as yet). Furthermore, this review included a large proportion of male subjects. Due to the clinical predominance of males within the BAV population, statistical analyses were adjusted for the differences in prevalence. Although these corrections have been made, the interpretation of these results for female subjects still should be done cautiously.

## Conclusion

The reported prevalence of CAD and CAD risk factors are comparable between BAV and TAV patients when adjusted for the age and sex differences between both groups.
